# Cutaneous Manifestations of Rheumatoid Arthritis: Diagnosis and Treatment

**DOI:** 10.3390/jpm13101479

**Published:** 2023-10-10

**Authors:** Michael J. Diaz, Nicole Natarelli, Aria Wei, Michaela Rechdan, Elizabeth Botto, Jasmine T. Tran, Mahtab Forouzandeh, Jose A. Plaza, Benjamin H. Kaffenberger

**Affiliations:** 1 College of Medicine, University of Florida, Gainesville, FL 32610, USA; 2 Morsani College of Medicine, University of South Florida, Tampa, FL 33602, USA; 3 School of Medicine, University of Texas Southwestern Medical Center, Dallas, TX 75390, USA; 4 School of Medicine, Indiana University, Indianapolis, IN 46202, USA; 5 Department of Dermatology, University of Florida, Gainesville, FL 32606, USA; 6 Department of Dermatology, Ohio State University Wexner Medical Center, Columbus, OH 43221, USA

**Keywords:** rheumatoid arthritis, cutaneous manifestations, vasculopathies, rheumatoid nodules, neutrophilic dermatoses, sweet syndrome, pyoderma gangrenosum, histopathology, quality of life

## Abstract

Rheumatoid arthritis (RA) is a chronic, systemic autoimmune disorder characterized by inflammatory arthritis and periarticular structural damage. Available evidence suggests that RA results from complex interactions between genetic susceptibility (e.g., HLA-DRB1), environmental factors (e.g., smoking), and immune dysregulation. Alongside joint-related symptoms, individuals with RA may also experience a wide array of skin issues, including the development of nodules, neutrophilic dermatoses, vasculitis, and vasculopathy. Treatment strategies for these manifestations vary but routinely involve corticosteroids, disease-modifying anti-rheumatic drugs, and biologics, with individualized approaches guided by disease severity. In this review, we provide comprehensive insights into the skin-related issues associated with RA, outlining their clinical characteristics and histopathological findings. Our aim is to facilitate early diagnosis and personalized treatment to improve the quality of life of affected individuals.

## 1. Introduction

Rheumatoid arthritis (RA) is a chronic, systemic autoimmune disorder characterized by symmetrical synovial joint and extra-articular inflammation, leading to progressive joint and periarticular structural damage, functional disability, and reduced quality of life [1]. As an intricate interplay of immune dysregulation, RA affects not only synovial joints but also induces diverse cutaneous manifestations such as rheumatoid nodules, neutrophilic dermatoses, and vasculitis. Other extra-articular manifestations that parallel the systemic course of RA include lymphoma and non-melanoma skin cancer, interstitial lung disease, and cardiovascular complications [2]. Taken together, these symptoms significantly impact patient morbidity and mortality.

RA is a multifactorial disease involving a complex interplay between genetic susceptibility, environmental triggers, and immune dysregulation. The pathogenesis of RA involves the activation of immune cells, including T-lymphocytes, B-lymphocytes, and macrophages. Auto-reactive B-cells in RA are central proponents of the secretion of anti-citrullinated protein antibodies (ACPA), rheumatoid factors (RFs), and pro-inflammatory cytokines, contributing to the disease progression [3,4]. They also activate T-cells and produce cytokines that sustain inflammation. T-cells play a critical role in activating macrophages and fibroblasts, which—in conjunction with osteoclasts—contribute to synovial hyperplasia, angiogenesis, and bone erosion. T-helper (Th) cells, including subsets such as Th1, Th2, Th17, and regulatory T (Treg) cells, produce pro-inflammatory cytokines like tumor necrosis factor-alpha (TNF-α), interleukin-6 (IL-6), and interleukin-17 (IL-17). These cytokines contribute to synovial inflammation, pannus formation, and subsequent cartilage and bone weakening [4]. Furthermore, other immune cells—including mast cells, dendritic cells, and natural killer cells—are also implicated in RA immunopathogenesis. The concerted actions of these immune cell populations induce an additional inflammatory response, tissue damage, and perpetuation of the disease through the release of antigen presentation, inflammatory mediators, and cytotoxic activity [4,5].

A number of genetic factors have been linked to an increased risk of RA, including the human leukocyte antigen (HLA)-DRB1 gene and specific polymorphisms [6]. The condition predominantly affects women, with a female-to-male ratio of 3 to 1. While the reason for the gender disparity is not fully understood, it also affects women at a younger age, often between 20 and 40 years old, whereas men are more likely to be affected in later years [7]. Smoking is the most significant environmental risk factor associated with RA, potentially contributing to RA- or RA-related autoimmunity development through complex biological interactions between ANCA-positive or RF factors [6].

The management of RA involves a multidisciplinary approach, including pharmacological interventions and physical therapy. While there is no cure currently available, the goals of treatment are to reduce joint inflammation, maximize functional capability, and prevent further joint damage. First-line management consists of nonsteroidal anti-inflammatory drugs (NSAIDs) and corticosteroids. Corticosteroids are indicated for short-term use at low doses during exacerbations or flares due to their ability to inhibit the release of phospholipids and reduce the activity of eosinophils, effectively decreasing inflammation. While corticosteroids are a more potent anti-inflammatory agent, NSAIDs offer the advantage of having fewer side effects [8]. The second-line treatment strategy for RA is aimed at achieving remission by stopping the advancement of joint damage and deformity. Disease-modifying anti-rheumatic drugs (DMARDs), a class of medications that target immune dysregulation and inflammation, such as hydroxychloroquine, methotrexate, leflunomide, and sulfasalazine form the cornerstone of second-line pharmacotherapy for RA [9]. Biological DMARDs, such as TNF inhibitors, interleukin inhibitors, and Janus kinase inhibitors, have revolutionized the treatment of RA by preventing the recruitment of cells that induce intracellular inflammation [9]. However, authors suggest DMARDs, such as etanercept—a recombinant human soluble TNF receptor—may be less efficacious in the treatment of the extraarticular features of RA, such as tenosynovitis and rheumatoid nodules [10]. Furthermore, the use of immunomodulatory agents has been used therapeutically to suppress the immune system’s abnormal response, thereby reducing both joint and skin inflammation [11]. As the understanding of RA’s immunopathogenesis deepens, so does the need for targeted interventions that encompass the complexities of both joint and skin involvement. This review aims to summarize the range of skin manifestations in rheumatoid arthritis and assess the available diagnostic and therapeutic approaches based on current evidence.

## 2. Methods

An initial literature search was conducted in July 2023 using the PubMed, MEDLINE, and Scopus databases (since inception) for original articles, systematic reviews, case series, and reports related to rheumatoid arthritis and its dermatologic manifestations using keywords such as “rheumatoid arthritis” and “cutaneous manifestation”. Reference chaining and advanced keyword searches conducted until August 2023 were used to identify additional records. Articles were selected and screened primarily by three reviewers (N.N., A.W., and E.B.). Authors M.J.D. and J.T.T. resolved article selection disputes. A total of 88 articles were selected for narrative review.

## 3. Results

### 3.1. Nodules

#### 3.1.1. Rheumatoid Nodules

**Figure 1:**
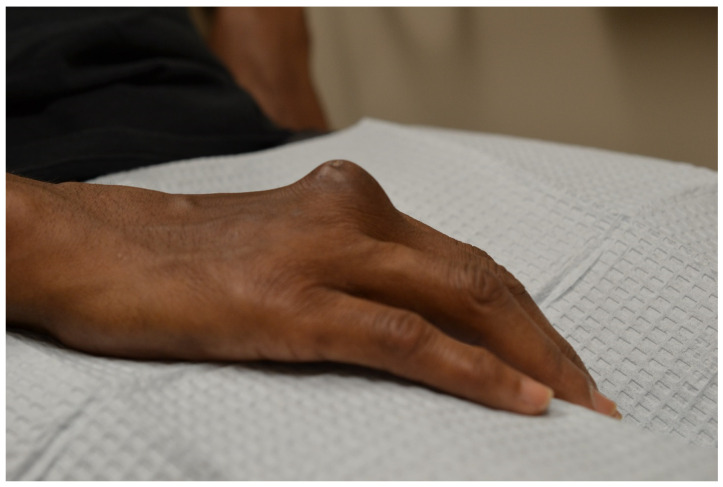
Rheumatoid nodule of the dorsal right hand.

**Figure 2:**
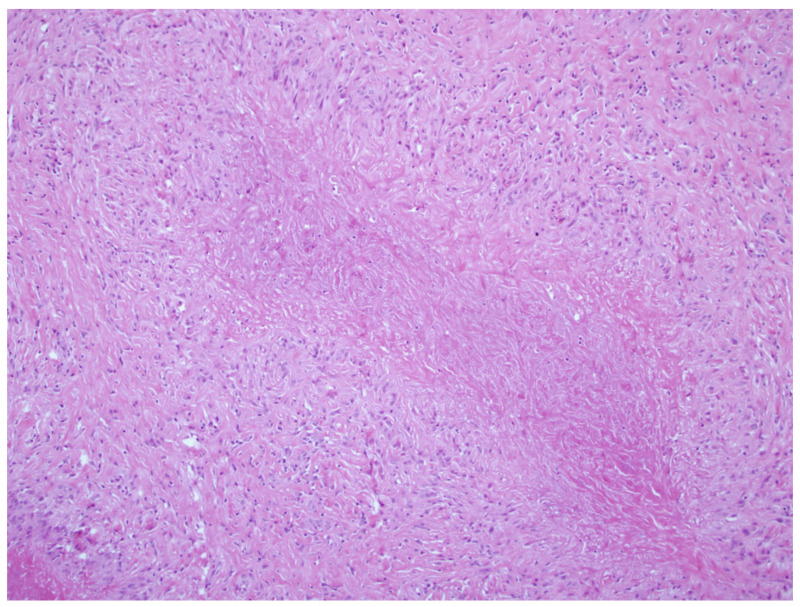
Rheumatoid nodule, H&E stain.

Rheumatoid nodules (RNs) are subcutaneous masses that manifest as one of the primary extra-articular features of severe rheumatoid arthritis (RA) and are commonly found alongside high levels of rheumatoid factor and antinuclear antibodies (Figure 1) [12]. These nodules typically form on extensor surfaces and regions subject to repetitive pressure, such as the joints of the hands, fingers, elbows, or feet (Table 1). Usually painless, these nodules occasionally become tender or inflamed following trauma, leading to potential complications like ulceration, infection, or rupture [13]. Nodule formation involves an abnormal immune-mediated accumulation of palisading macrophages and granulation tissue composed of lymphocytes and histiocytes, resulting in a central area of necrosis (Figure 2) [14]. About 20–30% of individuals with RA will develop these RNs, and their differential diagnosis includes rheumatoid nodulosis, foreign body granulomas, subcutaneous granuloma annulare, and subcutaneous nodules associated with rheumatic fever [15]. Interestingly, Langevitz and Urowitz found patients with a gold-induced adverse drug reaction were more likely to have nodular disease; therefore, nodules may be a predictor of adverse drug reactions [16]. Furthermore, accelerated nodulosis has been described with some medications indicated for the management of rheumatoid arthritis, such as methotrexate and azathioprine [17]. While RNs are commonly associated with RA, they are not exclusive to this condition and occur in other connective tissue diseases such as systemic lupus erythematosus, ankylosing spondylitis, and rheumatoid fever [18].

Managing RA symptoms can indirectly help treat rheumatoid nodules. However, certain disease-modifying anti-rheumatic drugs (DMARDs) like leflunomide and methotrexate, which are commonly prescribed to control inflammation and slow down RA progression, have been associated with the development of new RNs [19,20]. On a positive note, medications such as sulfasalazine, colchicine, penicillamine, tocilizumab, and rituximab have been reported to decrease the size and density of nodules. Moreover, corticosteroid injections administered directly into the nodules show promise in reducing inflammation and decreasing nodule size [21]. In cases where nodules cause significant pain, functional impairment, or nerve compression, surgical excision may be considered for more rapid treatment [22].

Currently, there are no major clinical trials specifically focused on the treatment of RNs. However, an ongoing study investigating the efficacy of tissue stromal vascular fraction in musculoskeletal pain, dysfunction, degeneration, or inflammatory disorders might have potential implications in the care of RNs [23].

#### 3.1.2. Rheumatoid Nodulosis

Rheumatoid nodulosis is a variant of RA that is associated with palindromic rheumatism, subcutaneous rheumatoid nodules, radiologic subchondral bone cysts, and a generally benign clinical course (Table 1) [24]. On histology, it closely resembles true RNs and shows a pattern of palisading necrotic granulomas [18]. This condition predominantly affects men aged 30–50 years and starts at a younger age than rheumatoid disease [25]. Benign rheumatoid nodulosis has also been reported in healthy children, with nodules commonly appearing over the prepatellar areas, pretibial areas, feet, scalp, and malleoli [26,27]. Unlike classic RA, the cystic lesions observed in rheumatoid nodulosis generally do not lead to erosive arthritis and tend to exhibit mild or absent systemic manifestations [28]. However, there have been rare cases where rheumatoid nodulosis presented as a form of RA, leading to severe joint damage over time [25]. The differential diagnosis for rheumatoid nodulosis is similar to that of true RNs and includes conditions such as necrobiosis lipoidica, rheumatic fever, sarcoidosis, systemic lupus erythematosus, subcutaneous granuloma annulare, and necrobiotic xanthogranuloma [24,29].

Due to its benign nature, rheumatoid nodulosis is self-limited and can be symptomatically controlled with NSAIDs and prednisone [25,28]. Subcutaneous injections of steroids have also proved to reduce the size of nodules [30]. When topical corticosteroids prove ineffective, topical tacrolimus has demonstrated efficacy in reducing nodule size and improving unwanted corticosteroid manifestations [31]. Hydroxychloroquine and pyridoxine are also effective treatments for rheumatoid nodulosis [32]. For complicated lesions that are numerous, larger, or adhere to bone, surgical resection is an option and is recommended for both functional and cosmetic reasons, although recurrence may be a concern [33,34]. Given the potential progression to classical RA in some patients, rheumatoid nodulosis requires prolonged follow-up and early treatment to prevent bone erosions and joint destruction [25]. To our knowledge, there are no major clinical trials currently underway for the treatment of rheumatoid nodulosis, nor are there adequate studies comparing the efficacy of treatment options.

#### 3.1.3. Accelerated Rheumatoid Nodulosis

Accelerated rheumatoid nodulosis was first characterized by the onset of new subcutaneous nodules in patients undergoing methotrexate (MTX) treatment for RA (Table 1) [35]. Further discovery found that approximately 12% of RA patients experience these nodules, which regress upon discontinuation of MTX. The timeline of accelerated rheumatoid nodulosis varies widely, occurring within 3 months to 12 years after initiating MTX with an average of around 3 years into the treatment [35,36]. On histology, the newly developed nodules exhibit similar features to typical RNs, such as palisading granulomas, giant cells, and fibrinoid necrosis [37]. However, accelerated rheumatoid nodulosis display distinct characteristics such as faster growth, a higher tendency to affect the hands, and an association with the HLA-DRB1*0401 allele [37,38]. Interestingly, this condition develops even when RA disease activity is well controlled and can occur without pre-existing RNs [38]. Since its initial discovery, accelerated rheumatoid nodulosis has also been linked to treatment with other medications such as leflunomide, etanercept, azathioprine, tocilizumab, letrozole, and infliximab anti-TNF-α therapy [35,39].

The primary treatment for accelerated rheumatoid nodulosis is discontinuing the drug that triggered its onset [35]. When this is not clinically feasible, drugs that inhibit the adenosine A1 receptor may be used. This receptor is stimulated by adenosine production from infiltrating monocytes, leading to giant cell formation and subsequent nodule development [40]. Certain medications like hydroxychloroquine, d-penicillamine, colchicine, and sulfasalazine have been shown to reduce the frequency of nodules [36]. However, given that accelerated rheumatoid nodulosis is associated with minimal discomfort or morbidity in a majority of cases, the continuation of the underlying drug may be an option if the newly developed nodules do not bother the patient and arthritis is adequately controlled [35,41]. To our knowledge, there are no major clinical trials currently underway for the treatment of accelerated rheumatoid nodulosis, nor are there adequate studies comparing the efficacy of treatment options.

**Table 1:** Clinical and histological characteristics of nodular manifestations.

Condition	Etiology	Histology	Affected Areas	Treatment
Rheumatoid nodules	-Positive RF -Smoking	-Palisading macrophages -Granulation tissue	-Hands, fingers, elbows, feet	-SSZ, TCZ, RIX, colchicine, DPA -Corticosteroid injections -Surgery
Accelerated rheumatoid nodulosis	-MTX use -HLA-DRB1*0401	-Palisading macrophages -Granulation tissue	-Hand, feet, ear	-Stop triggering drug -HCQ, DPA, SSZ, colchicine
Rheumatoid nodulosis	-No erosive arthritis -Negative RF	-Palisading macrophages -Granulation tissue	-Pretibial areas, feet, scalp, malleoli	-Prednisone, NSAIDs -Topical TAC, HCQ, DPA, PN -Surgery

RF = rheumatoid factor; MTX = methotrexate; SSZ = sulfasalazine; TCZ = tocilizumab, RIX = rituximab; HCQ = hydroxychloroquine; DPA = d-penicillamine; TAC = tacrolimus; PN = pyridoxine.

### 3.2. Neutrophilic Dermatoses 

Neutrophilic dermatoses are cutaneous lesions with histology depicting epidermal, dermal, or hypodermal neutrophilic infiltrates in the absence of infection or vasculitis [42]. Neutrophilic dermatoses associated with rheumatoid arthritis conventionally include Sweet syndrome, pyoderma gangrenosum, rheumatoid neutrophilic dermatitis, palisaded neutrophilic granulomatous dermatitis, and interstitial granulomatous dermatitis. As potential extra-articular manifestations of disease, the management of rheumatoid arthritis must include the management of such dermatoses. Of note, many overlapping features exist amongst the neutrophilic dermatoses, and many of them are associated with diseases other than RA. Because of these complexities, the literature lacks a demonstration of treatment outcomes and comparisons of treatment efficacies for many of the individual dermatoses.

#### 3.2.1. Sweet Syndrome

**Figure 3:**
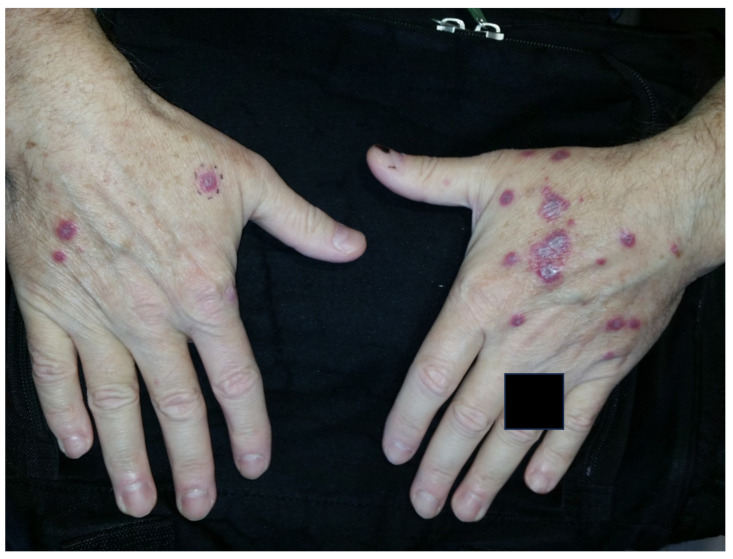
Sweet syndrome; neutrophilic dermatosis of the dorsal hands.

**Figure 4:**
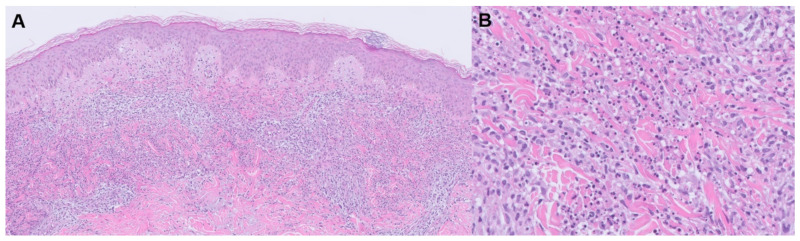
Sweet syndrome histology. (**A**) H&E stain; (**B**) magnified.

Sweet syndrome, also known as acute febrile neutrophilic dermatosis, is a disorder characterized by acute-onset tender plaques or nodules, fever, arthralgia, ophthalmologic manifestations, headaches, and sometimes oral or genital lesions (Figure 3 and Figure 4) (Table 2) [43]. In addition to rheumatoid arthritis, Sweet syndrome may occur in association with various other inflammatory and autoimmune diseases, such as inflammatory bowel disease, systemic lupus erythematosus, Sjogren syndrome, Hashimoto thyroiditis, Behcet disease, and dermatomyositis. Histology depicts a diffuse, dense neutrophilic infiltrate in the reticular dermis with interstitial leukocytoclastic nuclear debris. The epidermis is often spared, but spongiosis and subcorneal pustules may present [43]. The primary clinical differential diagnosis of Sweet syndrome is infection, although the histologic differential includes bowel bypass syndrome, erythema elevatum diutinum, granuloma faciale, halogenoderma, leukemia cutis, infection, leukocytoclastic vasculitis, lobular neutrophilic panniculitides, neutrophilic eccrine hidradenitis, pyoderma gangrenosum, and rheumatoid neutrophilic dermatitis [44].

If the lesions are limited, systemic or topical corticosteroids are the first-line treatment, with the goal of reducing morbidity and complications. Other first-line oral systemic agents include potassium iodide and colchicine, which may be useful in the case of corticosteroid contraindication [44]. Second-line treatments include indomethacin, cyclosporine, and dapsone. Additionally, reports have depicted the successful treatment of refractory cases with rituximab [45], anti-TNF-α agents [46], IL-1 receptor antagonists, [47,48], and granulocyte and monocyte adsorption apheresis [49]. While steroid-sparing agents have depicted efficacy, corticosteroids continue to be the first-line therapy for most patients with Sweet syndrome, including prednisone at a dose of 0.5–1 mg/kg/day [50], although dapsone is an effective alternative, especially in the hospital setting with neutropenic patients [51]. To our knowledge, there are no major clinical trials currently underway for the treatment of Sweet syndrome, however most cases are self-limited and resolve within 2–4 weeks of corticosteroid use in the absence of underlying malignancy or IBD [43].

#### 3.2.2. Pyoderma Gangrenosum

**Figure 5:**
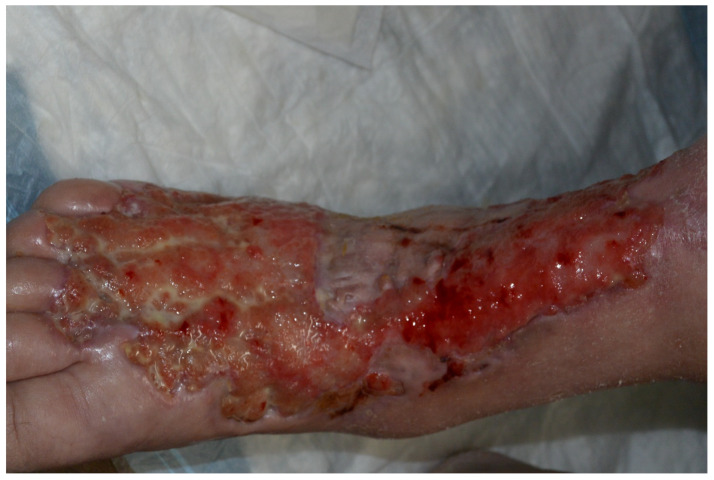
Pyoderma gangrenosum of the dorsal right foot.

**Figure 6:**
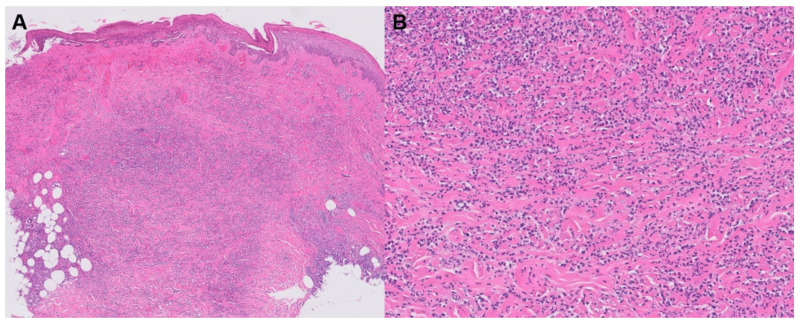
Pyoderma gangrenosum histology. (**A**) H&E stain; (**B**) magnified.

Pyoderma gangrenosum (PG) is a neutrophilic dermatosis presenting as painful skin ulcers with undermined borders and peripheral erythema (Figure 5) (Table 2) [52]. Lesions can rapidly progress to blistered or necrotic ulcers [53], necessitating appropriate wound care. Subtypes include “classical” PG, representing approximately 85% of cases, in addition to bullous, vegetative, pustular, peristomal, and superficial granulomatous variants [53]. Histology depicts an intense neutrophilic infiltrate with neutrophilic pustules and abscess formation (Figure 6). A differential diagnosis may include other causes of cutaneous ulceration, such as infections, malignancy, vascular ulceration, and systemic conditions such as systemic lupus erythematosus or Wegner’s granulomatosis. Biopsy can help exclude other pathologies, although it must be noted that Sweet syndrome and PG can coexist in the same patient [54].

The treatment of pyoderma gangrenosum is twofold, including anti-inflammatory therapies and proper wound care that fosters wound healing. A thorough laboratory assessment is required to ascertain the diagnosis and determine the underlying comorbidity driving the disease if not known [55,56]. First-line therapy consists of fast-acting immunosuppressive drugs, including topical or systemic corticosteroids and/or cyclosporine, depending on the extent of disease and targeting the underlying disease when known [52,57]. Prednisolone and cyclosporine showed equal effectiveness in PG ulcer healing, as well as the speed of healing, rate of recurrence, and association with adverse effects when compared in a randomized controlled trial in 2015 [58]. Use may be based on patient preference with consideration of side effect profiles. Long-term treatments include infliximab, other biologic TNF-α inhibitors, IL-12/23 inhibitors, and dapsone [56,59]. The first RCT of infliximab use for PG showed a beneficial clinical response in 69% of patients [60]. A small clinical trial from 2022 showed a complete re-epithelialization of target ulcers in 54.5% of patients with PG after 6 months of adalimumab therapy [61]. Other recent studies have shown TNF-α antagonists to induce a complete remission of PG refractory to first-line therapy in patients treated with infliximab (76%), adalimumab (64%), etanercept (47%) and certolizumab (100%) [62].

Intravenous immune globulin and alkylating agents can be used for refractory disease, especially when associated with hematologic disease. Complete or partial remission was achieved in 66.7% of PG patients who received IVIG in a retrospective cohort study [63] and in 88% of patients with refractory disease in a separate systemic review of cases [64]. In addition, wound care, which may include surgery in advanced cases, is essential to promote cutaneous healing.

Clinical trials are currently evaluating the efficacy of baricitinib, IFX-1, and Ixekizumab in the management of PG. Baricitinib is a JAK inhibitor with high affinity for JAK1 and JAK2 [65]. A phase II clinical trial with estimated completion in December, 2023 (NCT04901325) [66] is evaluating daily baricitinib and prednisone among 20 participants for 24 weeks, although preliminary results have yet to be shared. Another clinical trial (NCT03971643) [67] is assessing IV infusions of IFX-1 diluted in sodium chloride among 19 participants with PG. IFX-1 is an anti-C5a antibody that is hypothesized to block C5a-induced proinflammatory effects that may mediate PG development and progression. No results have been posted. Lastly, a phase II trial (NCT03137160) [68] evaluated 12 weeks of ixekizumab, an IL-17A inhibitor, dosed biweekly among four participants with PG. However, the trial was stopped prematurely due to safety, as three subjects developed serious adverse events including sepsis due to wound infection, severe sepsis secondary to pneumonia, and a bilateral infection of PG. 

At the time of writing, it remains difficult to comment on the treatment efficacy comparison for PG, as many modalities are analyzed in isolation or in small clusters, and RCTs are limited.

#### 3.2.3. Rheumatoid Neutrophilic Dermatitis

Characterized by erythematous papules, nodules, plaques, and urticaria-like lesions, rheumatoid neutrophilic dermatitis (RND) is a neutrophilic dermatosis with a prevalence of less than 2% among patients with rheumatoid arthritis (Table 2) [69]. RND typically occurs in patients with severe or active rheumatoid arthritis, often progressing years after the initial diagnosis of rheumatoid arthritis [69]. RND is histologically characterized by dermal neutrophilic infiltrate with variable degree of leukocytoclasis in the absence of vasculitis [70]. Clinically, the differential diagnosis includes other neutrophilic dermatoses such as Sweet syndrome and pyoderma gangrenosum, in addition to Behcet disease, bowel bypass syndrome, and rheumatoid nodules [71]. Histologically, RND can be distinguished from rheumatoid nodules, as the latter depicts palisading granulomatous inflammation with a necrotic center, palisading macrophages, and infiltrative inflammatory cells. Sweet syndrome, in contrast, is the most difficult to distinguish from RND based on similar histopathologic presentations [71].

Similar to Sweet syndrome, rheumatoid neutrophilic dermatitis can be managed with topical or systemic corticosteroids or other anti-neutrophilic therapies, such as dapsone dosed at 50–100 mg daily [70] or colchicine [72]. Cyclophosphamide and antimalarials such as hydroxychloroquine have also depicted efficacy [73]. Rheumatoid neutrophilic dermatitis lesions often resolve spontaneously or with an improvement of the underlying rheumatoid arthritis. However, an exacerbation of rheumatoid arthritis may promote lesion recurrence [72]. To our knowledge, the treatment of rheumatoid neutrophilic dermatitis is limited to case studies and small-scale reviews. Clinical trials are needed to assess and compare treatment outcomes [74].

#### 3.2.4. Palisaded Neutrophilic Granulomatous Dermatitis and Interstitial Granulomatous Dermatitis

**Figure 7:**
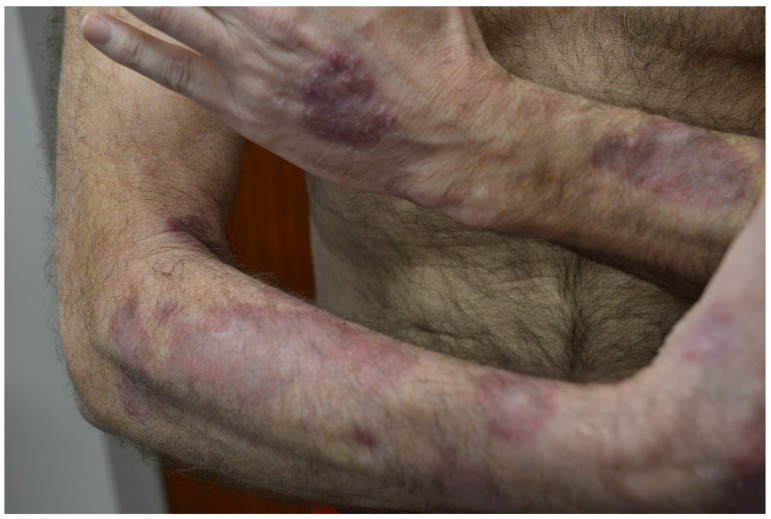
Palisaded neutrophilic granulomatous dermatitis of the dorsal arms.

**Figure 8:**
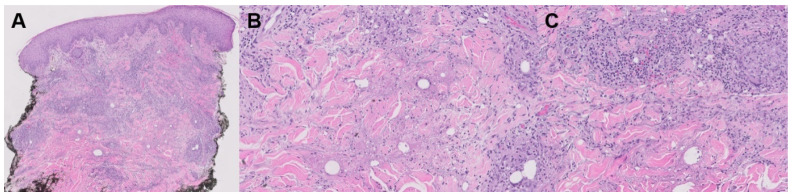
Palisaded neutrophilic granulomatous dermatitis histology. (**A**) H&E stain; (**B**,**C**) magnified.

Palisaded neutrophilic granulomatous dermatitis (PNGD) and interstitial granulomatous dermatitis (IGD) are both granulomatous dermatitis associated with systemic autoimmune disease (Table 2). PNGD presents with characteristic symmetric smooth, umbilicated, or crusted papules, often on the elbows and extremities with a skin to erythematous color (Figure 7) [75]. IGD shares many clinical features with PNGD, albeit with locations favoring the lateral trunk and skin folds [76]. While some clinicians consider PNGD and IGD distinct entities, they are often understood to exist on the same spectrum [77]. As such, “reactive granulomatous dermatitis” has been proposed as an encompassing term for both PNGD and IGD. Histologic findings differ slightly, with IGD characterized by lymphohistiocytic infiltrate with greater neutrophil infiltrate in PNGD (Figure 8) [78]. The differential diagnosis of PNGD on histology includes leukocytoclastic vasculitis, Sweet syndrome, and neutrophilic urticaria [79].

Treatments for both PNGD and IGD are often targeted at managing the underlying systemic condition. Clinical improvement of PNGD has been noted with topical or oral corticosteroids [80,81], oral dapsone [81,82], and hydroxychloroquine [75]. Similarly, clinical improvement of IGD has been observed with corticosteroids [83], dapsone [84], and hydroxychloroquine [85]. To our knowledge, no registered clinical trials currently exist for either PNGD or IGD, likely due to the rarity of these extraarticular manifestations of rheumatoid arthritis. Existing quantitative research on the efficacy of treatment modalities for individual neutrophilic dermatoses is also limited. Elsawi et al. recently assessed the use of IL-17 inhibitor for neutrophilic dermatoses and found that secukinumab was effective in 70% of analyzed studies [86].

**Table 2:** Clinical and histological characteristics, differential diagnosis, and treatment of neutrophilic dermatoses associated with rheumatoid arthritis.

Diagnosis	Clinical Characteristics	Histological Characteristics	Differential Diagnosis	Treatment
Sweet Syndrome	Acute-onset tender plaques or nodules, fever, arthralgia, ophthalmologic manifestations, headaches, oral or genital lesions	-Diffuse, dense neutrophilic infiltrate in the reticular dermis with interstitial leukocytoclastic nuclear debris -Epidermis often spared, but spongiosis and subcorneal pustules may be present	-Clinically: infection -Histologically: bowel bypass syndrome, erythema elevatum diutinum, granuloma faciale, halogenoderma, leukemia cutis, leukocytoclastic vasculitis, lobular neutrophilic panniculitides, neutrophilic eccrine hidradenitis, pyoderma gangrenosum, rheumatoid neutrophilic dermatitis	-First-line: systemic or topical corticosteroids, potassium iodide, colchicine -Second-line: indomethacin, clofazimine, cyclosporine, dapsone -Reported success in refractory cases: rituximab, anti-TNF-α agents, IL1 receptor antagonists, and granulocyte and monocyte adsorption apheresis
Pyoderma Gangrenosum	-Painful skin ulcers with undermined borders and peripheral erythema -Lesions can rapidly progress to blistered or necrotic ulcers	Intense neutrophilic infiltrate with neutrophilic pustules and abscess formation	Clinically: other causes of cutaneous ulceration including infections, malignancy, vascular ulceration, and systemic conditions such as systemic lupus erythematosus or Wegner’s granulomatosis -No definitive histopathological criteria	-Treatment includes anti-inflammatory therapies and proper wound care -First-line: fast-acting immunosuppressive drugs including topical or systemic corticosteroids and/or cyclosporine -Second-line: infliximab, other biologic TNF-α inhibitors, dapsone, minocycline -Refractory disease: IV immune globulin and alkylating agents
Rheumatoid neutrophilic dermatitis	Erythematous papules, nodules, plaques, and urticaria-like lesions	Dermal neutrophilic infiltrate with variable degree of leukocytoclasis in the absence of vasculitis	-Clinically: Sweet syndrome, pyoderma gangrenosum, Behcet disease, bowel bypass syndrome, rheumatoid nodules -Histologically: Sweet syndrome (difficult to distinguish based on similar histopathologic presentations)	-Topical or systemic corticosteroids, dapsone or colchicine -Efficacy demonstrated with cyclophosphamide and hydroxychloroquine
PNGD and IGD	-PNGD: symmetric smooth, umbilicated, or crusted papules, often on the elbows and extremities with skin to erythematous color -IGD: shared clinical features with PNGD, with locations favoring the lateral trunk and skin folds	-IGD: lympho-histiocytic infiltrate -PNGD: Greater neutrophil infiltrate	-PNGD, histologically: leukocytoclastic vasculitis, Sweet syndrome, neutrophilic urticaria	-Management of underlying condition -Noted clinical improvement with topical or oral corticosteroids, oral dapsone, and hydroxychloroquine

PNGD, palisaded neutrophilic granulomatous dermatitis; IGD, interstitial granulomatous dermatitis.

### 3.3. Vasculitis and Vasculopathy 

#### 3.3.1. Felty Syndrome

Felty syndrome is a more severe manifestation of rheumatoid arthritis with neutropenia and splenomegaly (Table 3). Disease progression follows a similar pattern to RA, is usually diagnosed in middle age, and preferentially affects females and Caucasians [87]. These patients usually have more joint inflammation and are prone to infections as a result of the splenic sequestration of neutrophils [88]. The dual nature of autoimmunity and immunocompromise in Felty syndrome makes it challenging to treat, with mortality as high as 25% [89].

Treatment for Felty syndrome is focused on treating the underlying RA and preventing infection. Currently, there are no randomized control studies evaluating treatment modalities. First-line treatment has historically involved the use of methotrexate or glucocorticoids [90]. Targeted immunotherapy has gained attention as of late; however, evidence of effectiveness is limited to observational studies [91]. A study in 2020 showed Tocilizumab, a monoclonal antibody, to successfully treat an FS patient, including the resolution of splenomegaly and stabilization of neutrophil count with no signs of development of infection [92]. A systematic review in 2017 identified numerous case reports that showed the success of Rituximab, another monoclonal antibody, in treating FS patients [93]. Other modalities include IVIG adjuvant therapy, described in a 2021 case report to have successfully resolved neutropenia and related infections. Splenectomy is another treatment option, shown to improve hematological response by 80% in FS patients who had recurrent infections or neutropenia [94]. The rarity of FS poses a challenge to advances in research exploring the efficacy of therapeutics for FS.

#### 3.3.2. Rheumatoid Vasculitis

**Figure 9:**
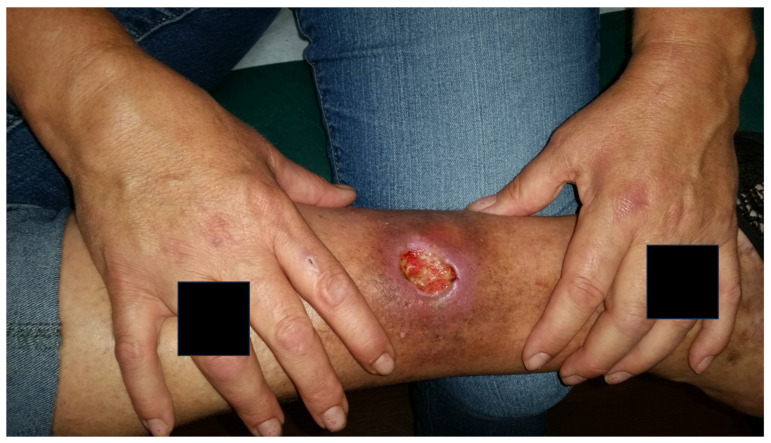
Rheumatoid vasculitis of the medial right leg.

Rheumatoid vasculitis is a rare but severe extra-articular complication of RA in which medium-sized arterioles supplying the skin and other organs are infiltrated within the muscular wall, resulting in vessel rupture (Figure 9) (Table 3). Focus on treatment is important, as the mean 5-year mortality rate is 30–50% [95], and this disease is typically associated with extremely elevated rheumatoid factor and poorly controlled disease.

Because of its severe nature, aggressive corticosteroid and immunosuppressive therapy has been the mainstay treatment for rheumatoid vasculitis [95]. Biologic agents are emerging as new treatment options, often as adjuvant therapy or after initial measures have failed [96]. Moreover, a smattering of evidence indicates that RV primarily limited to cutaneous manifestations does not respond as well to immunosuppressive therapies, further prompting the need for targeted biologics [96,97]. As with Felty Syndrome, more evidence is needed to establish standard treatment guidelines. Currently, treatment modalities rely on clinical success found in observational studies such as case reports or reviews. de Cerqueira et al. described the success of biologic agents including rituximab, infliximab, and etanercept used to treat RV [95]. Overall, clinical improvement was achieved by these modalities, including a decreased need for corticosteroids and even complete remission in 70% of the cases [95]. Rituximab has been used for the successful treatment of RV over the years, as supported by a 2020 retrospective study [98], which reported the complete remission of RV in 62% and partial remission in 38% of patients one year after initial treatment with Rituximab. At the time of writing, the newest biologic agents with reported clinical success in treating RV are tocilizumab and peficitinib [99].

**Table 3:** Vascular manifestations of rheumatoid arthritis.

Diagnosis	Characteristics	Clinical Findings	Differential Diagnosis	Treatment
Felty Syndrome	Severe RA features (erosive joint disease and deformity), Neutropenia, splenomegaly, vasculitis, necrotizing skin lesions, portal HTN, increased risk of malignancies, anemia of chronic disease, myeloid hyperplasia in bone marrow	Severe joint pain, swelling and deformities (esp. small joints of hands and wrists), fatigue, respiratory tract and skin bacterial infections, splenomegaly, variceal bleeding	SLE, large granular lymphocytic leukemia, other hematologic malignancies, drug reactions, amyloidosis, sarcoidosis, HIV, EBV	-MTX, glucocorticoids -Targeted immunotherapies: rituximab, tocilizumab -IVIG adjuvant therapy -Splenectomy
Rheumatoid Vasculitis	Severe RA features, cutaneous vasculitis, medium-sized artery necrotizing vasculitis, vasculitic peripheral neuropathy, ocular disease, cardiac disease, low serum complement C3	RA symptoms, deep leg ulcers, palpable purpura, rash, sores around nails, constitutional symptoms (fever, fatigue, weight loss), sensory neuropathy, visual disturbances	-Non-vasculitic RA cutaneous syndromes: Sweet syndrome, pyoderma gangrenosum -Other vasculitides: PAN, ANCA-associated, cryoglobulinemic, paraneoplastic -Vasculitis mimics: Infection, malignancy, endocarditis, thromboembolic diseases	-Corticosteroids and immunosuppressive therapy -Targeted immunotherapies: rituximab, infliximab, etanercept, tocilizumab, peficitinib

MTX, methotrexate; SLE, systemic lupus erythematosus; PAN, polyarteritis nodosa.

## 4. Conclusions

This review serves as an updated compendium on the typical cutaneous findings associated with rheumatoid arthritis, with a commitment to emerging evidence. Beyond their diagnostic value, these manifestations may serve as clinical indicators of disease severity and activity, aiding in treatment decisions and patient care. It is critical for physicians and other clinicians to be aware of these cutaneous features of the disease and specially to recognize more severe variants such as pyoderma gangrenosum and rheumatoid vasculitis from the generally indolent features such as rheumatoid nodules.

## Data Availability

Not applicable.
